# Video-Assisted Thoracoscopic Surgery (VATS) for Spontaneous Pneumothorax and Emphysematous Bullous Lung Disease: A Study From Northern India

**DOI:** 10.7759/cureus.25769

**Published:** 2022-06-08

**Authors:** Naveen Saini, Amandeep Nar, Harmandeep S Jabbal, Atul Mishra, Manavdeep S Bains

**Affiliations:** 1 Department of General Surgery, Manipal Hospital, Dwarka, New Delhi, IND; 2 Department of General Surgery, Dayanand Medical College and Hospital, Ludhiana, IND; 3 General & Minimal Access Surgery, All India Institute of Medical Sciences, Bathinda, IND; 4 Department of Surgery, Dayanand Medical College and Hospital, Ludhiana, IND; 5 Department of General Surgery, Bains Surgical & General Hospital, Nawanshahar, IND

**Keywords:** wedge resection, lobectomy, segmentectomy, bullectomy, broncho-pleural fistula, bpf, bullous lung disease, bld, video assisted thoracoscopic surgery, vats

## Abstract

Introduction: Bullous lung disease is the most common cause of spontaneous pneumothorax. The management of the same is primarily surgical, aiming at the bullectomy, which was earlier performed by standard postero-lateral thoracotomy. The last two decades have seen more frequent use of video-assisted thoracoscopic surgery (VATS) for the same and has been shown to be a low morbidity, efficacious, and cost-effective method. In this study we assess the role of VATS in the spectrum of bullous lung disease.

Method: The study was conducted in the Department of Surgery, Dayanand Medical College and Hospital, Ludhiana, for a period of three years from January 1, 2016 to December 31, 2019 in which patients with bullous lung diseases were enrolled and the role of video-assisted thoracoscopic surgery was assessed in them.

Results: The study included a total of 75 patients who were managed operatively either by VATS or open thoracotomy. In the study group, the average age of patients was 35.6 years (range 16-68 years). The most common presentation was only bullous lung disease (BLD) in 40% of patients followed by 32% of patients having both BLD and broncho-pleural fistula (BPF). Apical segmentectomy/non-anatomical wedge resection was done in 36% of patients whereas VATS bullectomy was done in 36% of patients. Elective conversion to thoracotomy was planned in six patients because of dense adhesions and thick pleural peel. We performed pleurodesis in almost all cases (96%). Mean blood loss in the VATS procedure was 48.7 ml and mean operative time was 67.2 minutes. Mean duration of hospital stay was 4.8 days. Forced expiratory volume (FEV1) increased significantly from a mean of 65.80% to 77.60%. There was significant increase in forced vital capacity (FVC) mean from 70.30% to 79.50%.

Conclusion: VATS can be used as a safe, feasible and effective procedure in patients presenting with spontaneous pneumothorax and bullous lung disease with or without a broncho-pleural fistula or parenchymal leak. It should be preferred over the traditional open thoracotomy procedure, whenever feasible to do so, in view of decreased perioperative morbidity and better functional outcome.

## Introduction

Bullous lung disease (BLD) presents usually with spontaneous pneumothorax, which is the accumulation of air within the pleural space that causes the lung to collapse. The underlying cause can be primary, such as the rupture of minor blebs or bullae in a patient who has no prior lung disease, or secondary, due to the existence of underlying lung disease. In either case, the basic goal of treatment is to eliminate intrapleural air collection and to prevent recurrence. This can be achieved by chest tube drainage in approximately 50% of cases [1]. The remaining cases require some form of surgical intervention.

Spontaneous pneumothorax due to rupture of bulla is quite common in otherwise healthy individuals. Bulla is an air-containing space within the lung that is formed by dilatation, destruction and confluences of air spaces distal to terminal bronchioles and is greater than 1 cm in diameter [2]. Surgical management aiming at the bullectomy was earlier performed by either standard posterolateral thoracotomy or, more frequently, smaller incisions [3]. But the last two decades have seen more frequent use of video-assisted thoracoscopic surgery (VATS) for the same and it has been proved to be a safe, effective, and cost-efficient procedure with low morbidity [4].

In this prospective observational study, the safety, feasibility and efficacy of VATS in patients with pneumothorax and bullous lung diseases are presented and evaluated.

## Materials and methods

The prospective observational study was carried out in the Department of General Surgery, Dayanand Medical College & Hospital, Ludhiana for a period of three years from January 1, 2016 to December 31, 2019 in which patients with pneumothorax and bullous lung disease were enrolled and the role of video-assisted thoracoscopic surgery was assessed in them.

Following written informed consent, the patient's information was recorded, and a full history was gathered, including symptoms, coexisting comorbid disorders, personal habits such as smoking or alcohol usage, and previous treatment history. All patients received a thorough examination that included all normal tests as well as a chest computed tomography. The various socio-demographic and clinical variables were noted. Patients were taken up for VATS after a thorough pre-anesthetic examination and given informed consent.

Inclusion criteria

Inclusion criteria were: Patients diagnosed on CT chest to have symptomatic bullous lung disease; Patients with symptoms related to giant bulla/bullae like chest pain, dyspnea, hemodynamic dysfunction; and Symptomatic bullous emphysematous lesions with normal intervening lung (symptoms like dyspnea, chest pain).

Exclusion criteria

People having the following contraindications to VATS were excluded: markedly unstable or patients in shock; extensive adhesions obliterating pleural space; prior talc pleurodesis; inability to tolerate single lung ventilation; and bleeding disorders.

People having the following contraindications to bullectomy were excluded: multiple small bullae (poorly defined bullae on chest imaging); severe chronic obstructive pulmonary disease (COPD); respiratory failure; and corpulmonale/pulmonary hypertension.

Surgical procedure

A patient with bullous lung disease was taken up for VATS after clearance from the anesthesia team. After induction of anesthesia and placement of foley’s catheter, patient was positioned laterally with the affected side above. Patient’s position was secured with belts to prevent displacement. The steps are as described by Ng et al. [5].

1. Port Placement

VATS was performed using the three-port method. For the insertion of a 0-30 degree telescope, the thoracoscope port was inserted at the seventh or eighth intercostal spaces along the mid-axillary line. Additional ports were inserted under direct thoracoscopic view with good triangulation.

2. Exploration

After a careful examination of the entire hemithorax, the region with the bulla was identified. The large bullae were punctured and collapsed to improve the thoracoscopic view, allow for easy instrumentation, and circumscribe the borders of the bullae from healthy lung tissue. Pleural adhesions, if present, were removed to allow for complete lung collapse and a good operating field. Adequate traction and hemostasis are critical during adhesiolysis.

3. Inferior Pulmonary Ligament Release

The inferior pulmonary ligament was released all the way down to the inferior pulmonary vein. Adequate traction of the inferior part of the lower lobe with atraumatic instruments, as well as the use of mounted swabs for blunt dissection, aided this process significantly. This allows for a better lung expansion in the post-operative period.

4. Resection of Bullae

**Figure 1:**
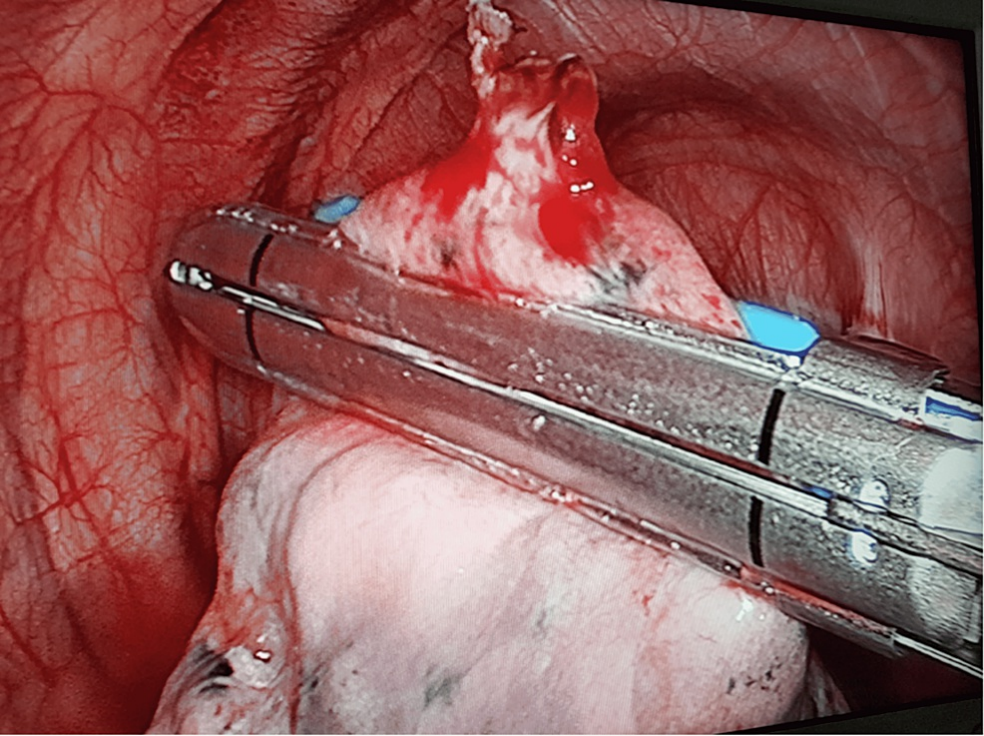
Video-assisted thoracoscopic surgery (VATS) bullectomy

**Figure 2:**
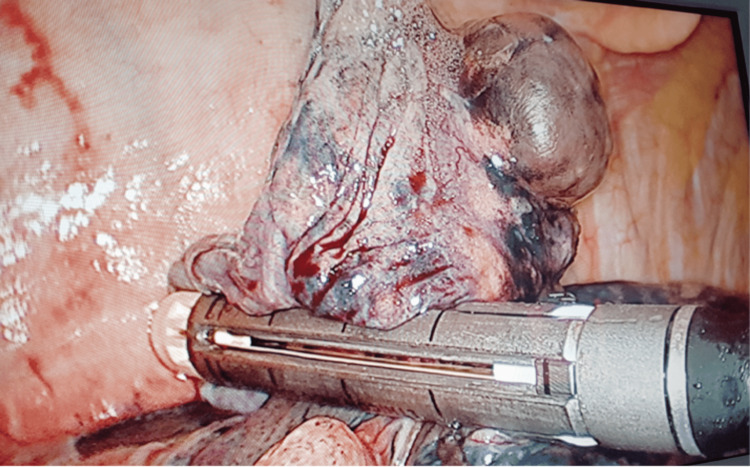
Video-assisted thoracoscopic surgery (VATS) wedge resection (non-anatomical)

Following delineation of the borders of the bullous segment, VATS bullectomy (Figure 1) was performed. Apical segmentectomy or non-anatomical wedge resection (Figure 2) was performed when multiple bullae were found in the apical segment across relatively healthier lung tissue using Blue 3.8mm height (for lung tissue), White 2.5mm height (for vessels), and Green 4.1mm height (for bronchus) cartridges or endoscopic suturing. Any persisting broncho-pleural fistula was repaired with endoscopic suturing if not resected.

5. Completion

The staple line, adhesiolysis areas, and port sites were checked for bleeding and hemostasis was established. Thorough lavage of the pleural cavity was done with warm saline. Chemical pleurodesis (3-5 g of graded talc) was done for larger bullae and BPF undergoing wedge resection/apical segmentectomy with BPF repair. Mechanical pleurodesis was performed for single apical bullae undergoing bullectomy [6-8]. Chest drains were inserted under direct thoracoscopic vision and connected to an underwater seal. The lung was then inflated under direct vision to check for any air leaks, and if any were discovered, fibrin glue was used to seal the leak. The operation was completed with layered closure of the stab wounds. Postoperative partial lung expansion on chest x-ray was addressed with a low-pressure negative suction device.

The final outcome was noted in view of symptomatic improvement, cure, recurrence of symptoms, complications, and conversion to open thoracotomy.

In this study we compared pre- and postoperative pulmonary function test, arterial blood gases, chest x ray and Hugh-Jones dyspnea grading in all patients. Patients presenting with pneumothorax were initially treated with a tube thoracostomy to relieve the pneumothorax and further subjected to the above-mentioned investigations.

Other parameters like operative time, intraoperative blood loss, duration of air leak and chest drains, duration of negative suction, postoperative hospital stay, early and chronic pain (for more than three months) and return to normal physical activity and work was also considered.

Outcome

Patients fulfilling the inclusion criteria were evaluated prospectively with a period of three months follow-up and final outcome was noted in view of the following: 1. Symptomatic improvement - improvement of dyspnea, improvement of hemodynamic function and pain relief; 2. Cure - no pneumothorax, full expansion lung and no bullae on chest imaging, no symptoms; 3. Complications - persistent air leak, bleeding from pulmonary vessels, intercostal nerve damage, postoperative re-expansion pulmonary edema, respiratory insufficiency, tumor implantation following VATS.

Statistical analysis

The data was recorded and frequency distribution was made and descriptive analysis was done. Chi-square/Fischer exact tests were used for association. P-value of < 0.05 was considered significant. The statistical analysis was done using Stata 9.2 licensed software (StataCorp., College Station, TX, USA).

The study was conducted following IRB approval from the Research and Ethics Committee, Dayanand Medical College and Hospital, Ludhiana.

## Results

The study included a total of 75 patients. Sixty-nine cases were managed operatively for spontaneous pneumothorax and bullous lung disease by VATS (92%). Remaining six cases underwent conversion from VATS to open thoracotomy (8%) due to dense adhesions and thick pleural peel (Table 1).

**Table 1:** Distribution of cases according to surgical approach

Procedure	No. of cases
Video-assisted thoracoscopic surgery (VATS)	69 (92%)
Open thoracotomy	6 (8%)

In the study group, the average age of patients was 35.6 years (range 16-68 years). There were 66 (88%) males and nine (12%) females. The minimum and maximum height were 157cm and 182cm respectively with mean of 172.2cm.

**Figure 3:**
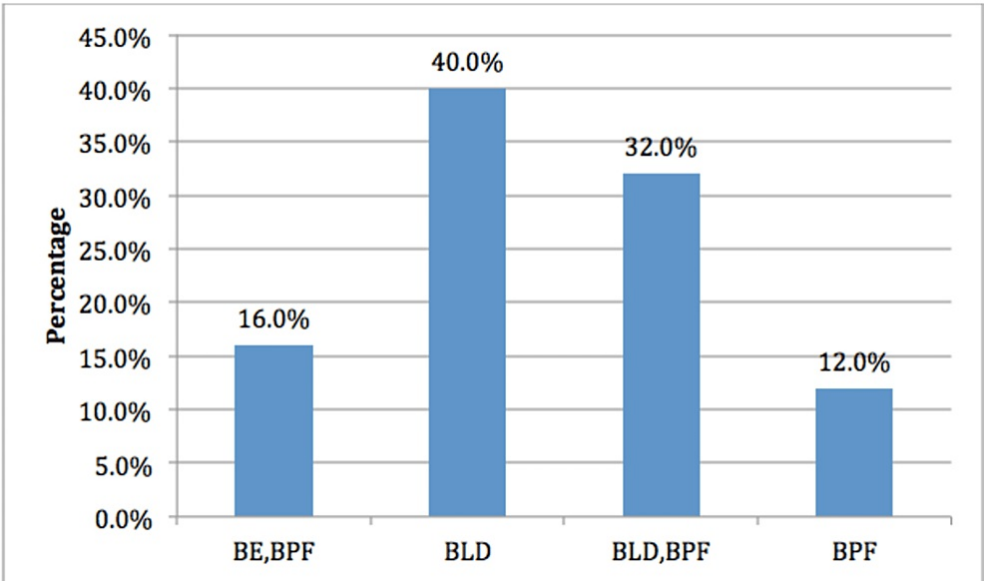
Distribution of patients according to the diagnosis BE: Bullous Emphysema; BPF: Broncho-Pleural Fistula; BLD: Bullous Lung Disease

Forty percent of patients were diagnosed to be having bullous lung disease (BLD) alone, 32% of patients had both bullous lung disease and broncho pleural fistula, 16% had bullous emphysema (BE) and broncho pleural fistula (BPF) and 12% had only broncho pleural fistula (Figure 3). Broncho-pleural fistula is defined as any pathological communication between the bronchial tree and pleural space. Minor parenchymal leaks are better described as alveolar-pleural fistulas (APF).

Depending upon the surgical procedure performed, apical segmentectomy/non-anatomical wedge resection was done in 36% of patients whereas VATS bullectomy was done in 36% of patients. Twelve percent of patients underwent BPF repair along with VATS bullectomy and 8% patients had VATS BPF repair only. Out of these, one underwent apical segmentectomy whereas BPF repair with decortication was done in second patient. Staplers were used in all 75 patients. Pleurodesis was done in 72 (96%) patients. However, it was not done in three patients due to presence of thick infected pleural peel.

Mean blood loss in VATS procedure was 48.7 ml and mean operative time was 67.2 minutes. Mean duration of hospital stay was 4.8 days (SD 1.8). The average days for return to normal activity after VATS procedure was seven days.

Postoperatively, prolonged air leak i.e. air leak more than seven days was observed in three patients (4%). Postoperative negative suction was applied in 21 patients for mean duration of four days (three to seven days). Out of these, six patients had partial lung expansion on postoperative chest x-ray whereas 15 had persistent air leak. Visual Analogue Scale (VAS) was used to quantify pain postoperatively. Pain decreased significantly in the postoperative period with mean decreased from 6.3 on postoperative day one to 2.4 on postoperative day three to 0.4 on postoperative day seven with p-value of <0.001. There was no pain at one month and three months of follow-up.

FEV1 increased from mean 65.80% to mean 77.60% of percentage predicted which is significant with p-value of <0.001. There was a significant increase in forced vital capacity (FVC), which increased from mean of 70.30% to mean of 79.50% of percentage predicted with p-value of <0.001 (Table 2). 

**Table 2:** Comparison of preoperative and postoperative mean fev1% predicted and mean fvc% of the predicted FEV: Forced Expiratory Volume; FVC: Forced Vital Capacity

	Pre-Op		Post-Op		p-value
	Mean	SD	Mean	SD	
FEV1 %PREDICTED	65.80	9.76	77.60	10.06	<0.001
FVC % PREDICTED	70.30	6.86	79.50	3.92	<0.001

Mean FEV1/FVC ratio increased from mean 78.44 to mean 87.34 in our study of 23 patients who underwent VATS procedure which is significant increase with p-value of 0.018.

**Figure 4:**
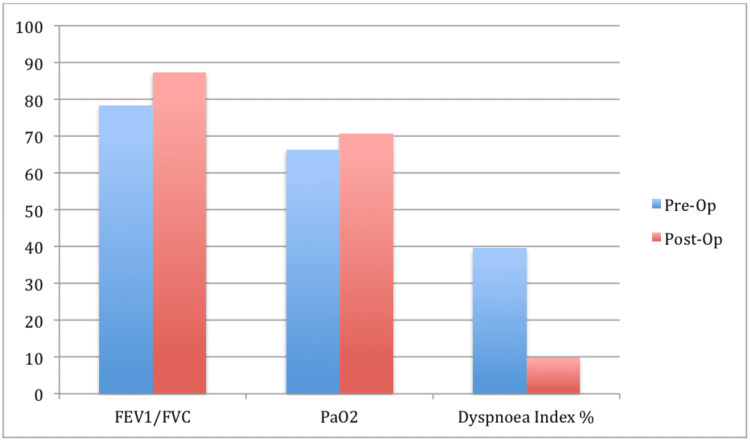
Comparison fev1/fvc; pao2; dyspnea index % in the patients before and after surgery FEV: Forced Expiratory Volume; FVC: Forced Vital Capacity, PaO2: Partial Pressure of Oxygen

Partial pressure of oxygen (PaO2) increased from mean of 66.35 mmHg to 70.70 mmHg difference, which was significant with p-value of <0.001. Partial pressure of carbon dioxide (PaCO2) decreased from 42.30 mmHg to 40.13 mmHg with p-value of <0.001 indicating significant decrease. Mean dyspnea index decreased from mean of 3.96 in the preoperative period to the mean of one at one-month postoperative follow-up with p-value <0.001 (Figure 4).

## Discussion

Bullous lung disease is one of the leading causes of spontaneous pneumothorax. Surgical intervention in the form of open thoracotomy has been used for decades to treat these patients; however, due to the morbidity associated with this procedure, both the patient as well as the referring pulmonologist is reluctant to choose this treatment modality. With advancements in minimally invasive equipment as well as techniques over the last two decades, traditionally performed open approaches, such as thoracotomy or sternotomy, have gradually been replaced by VATS [9].

VATS indications are diversifying as more experience is gained, and increased enthusiasm among surgeons has led to VATS being preferred over open thoracotomy these days. Despite better surgical outcomes and lower morbidity with VATS observed in various studies, there is still debate about the procedure's safety and efficacy. Over a three-year period, we assessed the safety, feasibility, and efficacy of video-assisted thoracoscopic surgery in the management of patients with bullous lung disease.

The majority of patients in the study were males (88%). The average age was 35.6 years and mean height was 172.2 cm. Inderbitzi and Furrer [10] also reported the incidence of bullous lung disease more in males as compared to females (70:30) with an average age of 36.5 years [10]. In a study by Lone et al. [11], mean height was 172.49+/-10.56 cm, a figure very close to as seen in our study.

Thoracoscopic diagnosis confirmed the presence of bullous lung disease in 40% of cases. The remaining 60% of cases characteristically had bronchopleural fistula or a parenchymal leak in addition to the underlying lung pathology. Inadequate management of the disease or late referral to the operating surgeon might be the reason behind this presentation. In addition, lack of surgical expertise and cost involved in dealing with such cases in our health system also leads to delay in surgical management of the patient.

The findings of decrease in complication rate and hospital stay observed in our study were similar to the results shown by most of the studies [12-14]. However, duration of surgery was longer in our study as compared to that by Inderbitzi and Furrer [10] because we included time from start of induction of anesthesia.

Our study showed that VATS offers early resumption of normal activities within seven days. This clearly demonstrates minimal physical and mental impairment following the VATS procedure.

Pain is an important factor in the postoperative period affecting the hospital stay, symptomatic relief and pulmonary function tests. Small incisions and less need for spreading ribs help in decreasing postoperative pain in patients undergoing VATS procedure as compared to open thoracotomy. Pain was assessed by VAS. Pain decreased significantly in the postoperative period with mean decreased from 6.3 on postoperative day one to mean of 2.4 on postoperative day two to mean of 0.4 on postoperative day seven. There was no pain at one month and three months of follow-up. Landreneau et al. [15] and Yim et al. [16] also showed significantly less postoperative pain in VATS group as compared to thoracotomy group.

Pulmonary function tests, PaO2 and PaCO2 were evaluated one month after surgery. Pulmonary function testing was done in 30 patients. Patients with bronchopleural fistula were excluded from pulmonary function testing. FEV1 increased from mean of 65.80 to mean of 77.60 of percentage predicted with p-value <0.001 indicating significant increase. FVC increased from 70.30 to 79.50 of percentage predicted which is a significant increase with p-value of 0.001. FEV1/FVC ratio also showed significant increase from 78.44 to 84.34 with p-value of 0.018. Schipper et al. [17] in their study showed increase in FEV1 from 34% of predicted value to 55% of the predicted value at six months of postoperative period. But they included only patients having giant emphysematous bullae in their study. This might be the reason for less increase of FEV1 in our study as compared to theirs. Palla et al. [18] also observed significant improvement in FEV1, FVC and FEV1/FVC values in postoperative period. PaO2 increased from mean of 66.35 mmHg to mean 70.70 mmHg postoperatively with p-value <0.001, which was a significant increase. PaCO2 also decreased significantly from mean of 42.30 mmHg to mean of 40.13 mmHg with p-value of 0.001. Similar results were reported by Schipper et al. [17]. Snider [19] also showed that hypoxemia and hypercapnia were frequently improved after surgery and FEV1 increased moderately.

There was a significant improvement in dyspnea index. Mean dyspnea index decreased from mean of 3.96 in the preoperative period to the mean of one at one month postoperatively. Wang et al. [20] and Lone et al. [11] also showed significant improvement in dyspnea index following VATS bullectomy.

## Conclusions

VATS can be used as a safe, feasible and effective procedure in patients presenting with spontaneous pneumothorax and bullous lung disease with or without a broncho-pleural fistula or parenchymal leak. When possible, it should be preferred over open thoracotomy due to lower perioperative morbidity and better functional outcome.
